# Supplementary material for: Patients’ E-Readiness to use E-Health technologies for oral health
Source: PLoS One. 2021 Jul 12;16(7):e0253922. doi: 10.1371/journal.pone.0253922 (PMC8274877; doi:10.1371/journal.pone.0253922)
Supplement: S1 File — (DOCX) [file pone.0253922.s001.docx]

**INTERVIEW GUIDE**

**Date:**

**Time and Place:**

**Number of Interview:**

1. **INTERVIEW OPENING**

- Greet and welcome the interviewee; introduce yourself (interviewer/position) and make the applicant feel comfortable
- Explain the purpose of the interview and inform the candidate about the duration of the interview; allow interviewee to read the consent
- Invite the interviewee to clarify any doubts regarding the interview and sign 2 copies of the consent form.
- Describe the interview process.
- Before starting, mention confidentiality to participant

Mention that:

- - - Your anonymity will be respected
    - Your name will not be revealed or published in any document.
    - You can withdraw at any time (if you feel uncomfortable)
    - Install the tape recorder (carry out a test recording)

**B) CONDUCTING THE INTERVIEW**

- Verify that the tape recorder is functioning correctly
- Begin with an unstructured, open questions to encourage spontaneity.
- Give a brief description at the end of each section to make sure that the participant understood well and to give him/her the opportunity to complete or modify their statements (reformulation).

**Section 1: core readinesS**

(*Gauges the extents to which members of a community are dissatisfied with the current status of their healthcare service provision, see e-health as a solution, and express their need and preparedness for e-health services*.)

1. How satisfied are you with your access to the oral healthcare system?
2. What are, in your opinion, the problems that you face in access to oral health care?
3. What are your expectations from the oral health care system?
4. How can access to oral health care be improved?
5. What came into your mind when you first heard about e-health technology?
6. Were you aware about e health technology?
7. Do you see e-health technologies (using the internet) as solution to these problems? Describe your point of view.
8. Have you tried using the internet to access health care? If yes, could you explain briefly? If no, what was the reason for your hesitation ?
9. Can you describe your needs in order for you to use e-oral health technologies?
10. How can the use of e-health technologies be improved?

**SECTION 2: ENGAGEMENT READINESS**

(Engagement readiness refers to “*Willingness to be trained, awareness and debating advantages and disadvantages”*

1. Are you willing to be trained to use e-oral health technology?
2. What factors would influence you in making the decision to use e-oral health technology?
3. What do you think about the advantages of e-oral health technology?
4. What do you think about the disadvantages of e-oral health technology?
5. What do you think would be the impact of e-oral technology on your oral-health?
6. How would you and society benefit from these technologies in access to e-oral health care?
7. In your opinion, how would e-oral technology change the way you access oral health care?
8. How would e-oral health technologies help in improving oral health care?

**SECTION 3: STRUCTURAL READINESS**

(Structural readiness is defined “*as the development of infrastructure such as adequate human resources, technical structures as well as necessary training for telehealth implementation*”)

1. What sources of technical knowledge, like internet connectivity, are available to you?
2. What facilities will motivate you to use e-oral health technology?
3. How can e-oral health implementation be made more convenient and better?
4. What norms, guidelines can make it more feasible?
5. What would be the impact of awareness programs, training and education on e-oral health technologies?
6. What barriers do you have regarding the use of these technologies?

**SECTION 4: NON-READINESS**

1. Are you prepared to participate in e-oral health technologies when it comes to access to oral health?
2. What, according to you, are the reasons for not being willing to use it?
3. Is there anything else that you would like to add?

**SECTION 5: PUBLIC READINESS**

(*Gauges the extent to which members of the public and patients are aware of, and can afford and access, e-health services*)

1. Within your financial capacity, is e-oral health affordable to you?
2. How convenient is it for you to access e-oral health?
3. How would you encourage a person from your community to use e-oral health technology?

**SECTION 6: SOCIO- DEMOGRAPHIC INFORMATION**

1. Tell me about yourself and your family?
2. How old are you?
3. Are you working presently?
4. What are your job responsibilities?
5. What is your marital status? Are you living alone?
6. Are you satisfied with your annual income?
7. Are you member or part of any social organisation or group?
8. Is there anything else that you want to add?
9. **Closing THE INTERVIEW**
10. Is there anything else that seems to you important and that you would like to talk about? any comment?
11. Thank the interviewee for his/her time and contribution
12. Ask if the participant can be contacted later, if necessary.
13. **AFTER THE INTERVIEW**
14. Write down any observations made during the interview.
